# Review of "*Haldane, Mayr, and Beanbag Genetics*" by Krishna Dronamraju

**DOI:** 10.1186/2041-2223-2-19

**Published:** 2011-09-19

**Authors:** Ken Weiss

**Affiliations:** 1 Evan Pugh Professor of Biological Anthropology and Genetics and Science, Technology, and Society, Department of Anthropology, The Pennsylvania State University, 409 Carpenter Building, University Park, PA 16802, USA

## Review

Krishna Dronamraju was the legendary population geneticist JBS Haldane's last student at the end of Haldane's life, when Haldane was working and teaching in India. Since then Dronamraju has made something of a career as a Haldane biographer, historian and nostalgist. He has written articles and books on Haldane's work and career, mixing the professional with the personal. This is another of that kind. It is a short, clearly written and interesting read.

The context is the discussion of the formative years of 20th-century genetics and evolutionary theory about how reductionist one can be to capture the nature of evolution and genetics effectively. The context is debate between Haldane and Ernst Mayr, whom the author also knew, about the relevance of the then-new field of population genetics. The phrase 'beanbag genetics' was coined in the mid-20th century to refer to an alleged view that evolution could be understood one gene at a time, each independently on its own terms, and that selection worked gene-by-gene. Mayr used the phrase to resist such reductionism, arguing that organisms rather than genomes are important, because they are the result of complex interactions among genes. The discussion took place as part of the formation of the 'modern synthesis' that united Darwin's and Mendel's thinking, a synthesis which began around 1920 and developed during the subsequent generation. Only by around the 1960s was it somewhat displaced theoretically by other issues, such as 'non-Darwinian' neutralism, and by a focus on the actual nature of genes and genomes that was being revealed.

Dronamraju argues that the disagreements between Mayr and Haldane were substantial, if gentlemanly, and their views were never as far apart as caricatures of them suggested. As Dronamraju points out, though their studies were largely single-gene-focused, population geneticists indeed considered multilocus interactive models, and their debate was not whether genes interact but how important nonadditive effects are in understanding the fate of variants at a single gene. The issues are alive today in the context of extensive sequence data, though few today pay much attention to these more theoretical details, being concerned instead, rightly or wrongly, with immediate analytical challenges rather than the role of history in framing our views today.

Dronamraju presents the arguments of the time and some of the subsequent views of and commentaries expressed by later population geneticists such as M Kimura and JF Crow. Classical experimental genetics was about identifying individual genes and their functions, in part because that was what one could theorize about specifically (and mathematically). But those researchers in classical 'quantitative' genetics were always interested in aggregate analysis based on measures such as heritability and genotypic variance rather than identification of individual genes and their contributions or their biological functions. Population genetics in essence dealt with changes in allele frequencies in a mathematically abstract way for which neither the form nor the function of a gene needs to be known.

Thus, there is little in this book that reflects the issues presented by modern high-throughput sequence data, by the use of genome-wide data in disease association studies, by expression array studies or by the relationship to signalling networks and the evolution of development. In this sense, the book is somewhat frozen in the time of a half-century ago, written from an historical retrospective view, and does not make a case for why or how these issues are important today rather than just 'dead history'. They are important, but in different ways. For example, questions are now being raised on the basis of new data about how function, fitness and population dynamics affect the structure of genomes, but those issues were beyond much discussion in the early days of genetics.

The book presents a brief biographical time line of the careers of both Haldane and Mayr, followed by about 66 pages of correspondence between Haldane and Mayr. These may be of some historical interest. Overall, this book is a cruise through mid-20th-century evolutionary genetics that gives the reader a taste of issues as they were debated when biology was grappling with the quantitative population problems presented by Darwin and Mendel in the previous century. Dr Dronamraju presents this debate to some extent in the context of his having been around personally at the time. While this book is not a meticulous history, especially with regard to technical details and relationships to modern issues, the kinds of disputes he chronicles provide an informative, useful and readable account of those formative times.

## Competing interests

The author declares that they have no competing interests.

